# The role of cholesterol and mitochondrial bioenergetics in activation of the inflammasome in IBD

**DOI:** 10.3389/fimmu.2022.1028953

**Published:** 2022-11-18

**Authors:** Jessica Astorga, Naschla Gasaly, Karen Dubois-Camacho, Marjorie De la Fuente, Glauben Landskron, Klaas Nico Faber, Félix A. Urra, Marcela A. Hermoso

**Affiliations:** ^1^ Laboratory of Innate Immunity, Program of Immunology, Institute of Biomedical Sciences, Faculty of Medicine, Universidad de Chile, Santiago, Chile; ^2^ Immunoendocrinology, Division of Medical Biology, Department of Pathology and Medical Biology, University Medical Center Groningen, Groningen, Netherlands; ^3^ Department of Gastroenterology and Hepatology, University Medical Center Groningen, Groningen, Netherlands; ^4^ Laboratory of Metabolic Plasticity and Bioenergetics, Program of Molecular and Clinical Pharmacology, Institute of Biomedical Sciences, Faculty of Medicine, Universidad de Chile, Santiago, Chile; ^5^ Laboratory of Biomedicine Research, School of Medicine, Universidad Finis Terrae, Santiago, Chile

**Keywords:** IBD - inflammatory bowel disease, intracellular cholesterol accumulation, mitochondrial dysfunction, inflammasome, NLRP3 inflammasome, diet phytochemicals

## Abstract

Inflammatory Bowel Disease (IBD) is characterized by a loss of intestinal barrier function caused by an aberrant interaction between the immune response and the gut microbiota. In IBD, imbalance in cholesterol homeostasis and mitochondrial bioenergetics have been identified as essential events for activating the inflammasome-mediated response. Mitochondrial alterations, such as reduced respiratory complex activities and reduced production of tricarboxylic acid (TCA) cycle intermediates (e.g., citric acid, fumarate, isocitric acid, malate, pyruvate, and succinate) have been described in *in vitro* and clinical studies. Under inflammatory conditions, mitochondrial architecture in intestinal epithelial cells is dysmorphic, with cristae destruction and high dynamin-related protein 1 (DRP1)-dependent fission. Likewise, these alterations in mitochondrial morphology and bioenergetics promote metabolic shifts towards glycolysis and down-regulation of antioxidant Nuclear erythroid 2-related factor 2 (Nrf2)/Peroxisome proliferator-activated receptor gamma coactivator-1 alpha (PGC-1α) signaling. Although the mechanisms underlying the mitochondrial dysfunction during mucosal inflammation are not fully understood at present, metabolic intermediates and cholesterol may act as signals activating the NLRP3 inflammasome in IBD. Notably, dietary phytochemicals exhibit protective effects against cholesterol imbalance and mitochondrial function alterations to maintain gastrointestinal mucosal renewal *in vitro* and *in vivo* conditions. Here, we discuss the role of cholesterol and mitochondrial metabolism in IBD, highlighting the therapeutic potential of dietary phytochemicals, restoring intestinal metabolism and function.

## Introduction

Inflammatory bowel disease (IBD), including ulcerative colitis (UC) and Crohn’s disease (CD), is characterized by a loss of intestinal barrier function and chronic inflammation of the gastrointestinal tract caused by an aberrant interaction between immune response and gut microbiota in genetically susceptible subjects (1–3).

IBD is a 21^st^ century global disease (4), with industrialized countries having a stable incidence (12 to 26 per 100,000 people) as well as a rising prevalence (5), based on genetic susceptibility, early diagnosis, and environmental factors, including diet, lifestyle, and comorbidities (6, 7). Systematic reviews of MEDLINE and EMBASE from 1990 until 2015 established the highest prevalence values for IBD in Europe (UC, 505 per 100,000 people in Norway; CD, 322 per 100,000 people in Germany) and North America (UC, 279 per 100,000 people in the USA; CD, 319 per 100,000 people in Canada) (4). In contrast, IBD incidence in newly industrialized countries, has increased with low prevalence, including countries in Asia, the Middle East, South America, and Africa (5).

The early diagnosis allows possibilities to treat short-term complications and reduce their severity to avoid hospitalization and surgery (8). Treatment possibilities for CD include corticosteroids, azathioprine, or immunomodulators such as methotrexate, while for UC, 5-aminosalicylates (5-ASA), corticosteroids, or azathioprine (9). Biologic drugs are currently used for treating severe to moderate IBD, such as anti-TNF blockers (infliximab, adalimumab, certolizumab pegol, and golimumab), enhancing intestinal mucosa healing, symptom relief, reduction in hospitalization rates, and improvement in quality of life (8). However, a third of patients are non-responsive to anti-TNF therapy or lose their response to treatment over time (10–12). Other biological drugs have been developed and used as primary therapy, or for those who failed anti-TNF therapy, such as anti-integrin agents (13). Nevertheless, these treatments as well as the hospitalization remain costly for IBD patients (14). Diet can be considered as a booster in the treatment of IBD patients, since some dietary components have been shown benefits preventing the onset of this disease (15).

Therefore, the innate immune system is essential for the first response of the host against infection or damage in the intestinal mucosa. Pattern Recognition Receptors (PRRs) recognize pathogens-associated molecular patterns (PAMPs) and/or damage-associated molecular patterns (DAMPs), such as Toll-like receptors (TLRs), nucleotide-binding oligomerization-domain protein-like receptors (NLRs), and C-type lectin receptors. There is evidence that inflammasome participates during IBD development, and its activation in the intestinal mucosa enhance the pathogen clearance (16, 17). The inflammasome is a supramolecular assembly recognizing PAMPs and/or DAMPs, serving as a platform for caspase-1 activation and production of mainly IL-1β and IL-18. The most studied inflammasome corresponds to the NLR family pyrin domain containing 3 (NLRP3), triggering intestinal mucosa inflammation. High IL-1β expression is related to lack of primary response to anti-TNF therapy. Therefore, NLRP3 inflammasome and IL-1β are crucial players during IBD development (18, 19).

Inflammasome activation requires two signals: the first is an NF-κB-mediated signal for *NLRP3* and pro-*IL1B* gene transcription. The second signal (required for its assembly) can be of diverse origins, including lipopolysaccharide (LPS), toxins, and viral and bacterial nucleic acid. Interestingly, second signals can also arise from cellular metabolism, such as excess intracellular cholesterol, oxidized low density lipoprotein (oxLDL), mitochondria-derived components (cardiolipin, reactive oxygen species (ROS), mitochondrial DNA), or endoplasmic reticulum (ER) stress, all currently related to IBD pathogenesis (20–23). This review summarizes the relevance of intracellular cholesterol changes and how alterations in mitochondrial bioenergetics influence inflammasome activation in IBD. Finally, the role of dietary compounds, especially phytochemicals, in the restitution of mitochondrial function and cholesterol homeostasis in this disease is considered.

## Alterations in gut barrier homeostasis and cell diversity during IBD

The intestinal barrier comprises the intestinal epithelium and mucus, coexisting with the lamina propria, the gut-associated lymphoid tissue (GALT), and the commensal gut microbiota (24). It allows digestion and absorption of dietary components and protects us against pathogens. A proper epithelial barrier function and an eubiotic gut microbiome are crucial for intestinal homeostasis (24, 25). Thus, during some gastrointestinal disorders, such as IBD, an imbalanced gut barrier appears, with increased pro-inflammatory mediators causing epithelial damage and, therefore, epithelial permeability, accompanied by an imbalanced gut microbiome, known as “gut dysbiosis” (24).

Located at the intestinal crypts, tissue-resident stem cells proliferate and differentiate into mature enterocytes, mucus-secreting goblet cells, or enteroendocrine cells, while migrating upwards through the crypt to the villus. Essential for adherens junctions between epithelial cells, tight junctions maintain intestinal barrier integrity, which can be disrupted by inflammation or gut dysbiosis (25). Tight junctions include adherens junctions, desmosomes, and gap junctions and they are formed by transmembrane proteins such as occludins, claudins, junctional adhesion molecules, tricelluin, and intracellular scaffolds proteins (zonula occludens (ZO) proteins). Intestinal stem cells have tight junctions distributed heterogeneously and different composition of transmembrane proteins, avoiding permeability closer to the crypt (26). Notably, UC is characterized by reduced epithelial cell diversity and decreased stem cell differentiation and migration, negatively affecting intestinal healing and mucus production by goblet cells (27).

Coordinating the immune response underneath the epithelial layer, immune cells (T cells, B cells, intraepithelial lymphocytes- IELs, and phagocytes) are organized in GALT, such as Peyer’s patches, mesenteric lymph nodes, and isolated lymphoid follicles (28).

Although, loss of gut homeostasis in IBD involves the participation of lamina propria and infiltrating immune cells, such as granulocytes, antigen-presenting cells (APCs), macrophages (Mø), B and T cells (29–31), as well as inflammatory cytokines, such as inflammasome-regulated IL-1β and IL-18, amongst others (TNF-α, IL-6, IL-12, IL-17, IFN-γ, GSDM (32, 33)). Despite an evident relationship between immune cells, metabolism, and inflammasome activation is expected, the role of metabolic mediators, such as cholesterol in IBD remains not fully understood.

## Intracellular cholesterol accumulation in IBD

### Cholesterol sources and homeostasis

Cholesterol is a sterol that occurs in two forms: free cholesterol and esterified cholesterol, the latter of which is a result of the conjugation of a cholesterol molecule to a fatty acid (FA, usually palmitic or oleic acid, and less frequently, linoleic acid) *via* an ester bond. Physiologically, cholesterol is an essential lipid of animal cell plasma membranes and a precursor of a variety of biologically active compounds, such as bile salts, hormones, and vitamin D3 (34). Additionally, cholesterol interacts with phospholipid FAs, decreasing the mobility of their hydrocarbon chains and consequently reducing the plasma membrane fluidity.

Dietary absorption (“*exogenous cholesterol*”) and endogenous synthesis (“*endogenous cholesterol*”) are the main sources of cholesterol in the body. Accordingly, principal cholesterol metabolic routes refer to its function in biosynthetic processes, excretion in the digestive tract, and an alternative route of removal, known as trans-intestinal C excretion (TICE) to complete the cholesterol balance (35). To maintain physiological cholesterol levels, also known as “*cholesterol homeostasis*”, all pathways are subjected to regulatory mechanisms responsible for cholesterol balance in the plasma membrane and intracellular.

*Endogenous cholesterol* is synthesized by a long and complex route, whose limiting enzyme HMG-CoA reductase (HMGCR) expression is activated by the sterol regulatory element-binding protein 2 (SREBP-2) transcription factor, localized in the ER (36).

Approximately 40-50% of *exogenous* cholesterol comprising the conversion of cholesterol to bile acids (BAs) and the excreted cholesterol *via* mixed micelles is reabsorbed through enterocyte pathways and once esterified, is incorporated in intestinal lipoproteins, named the chylomicrons (35).

**Figure 1:**
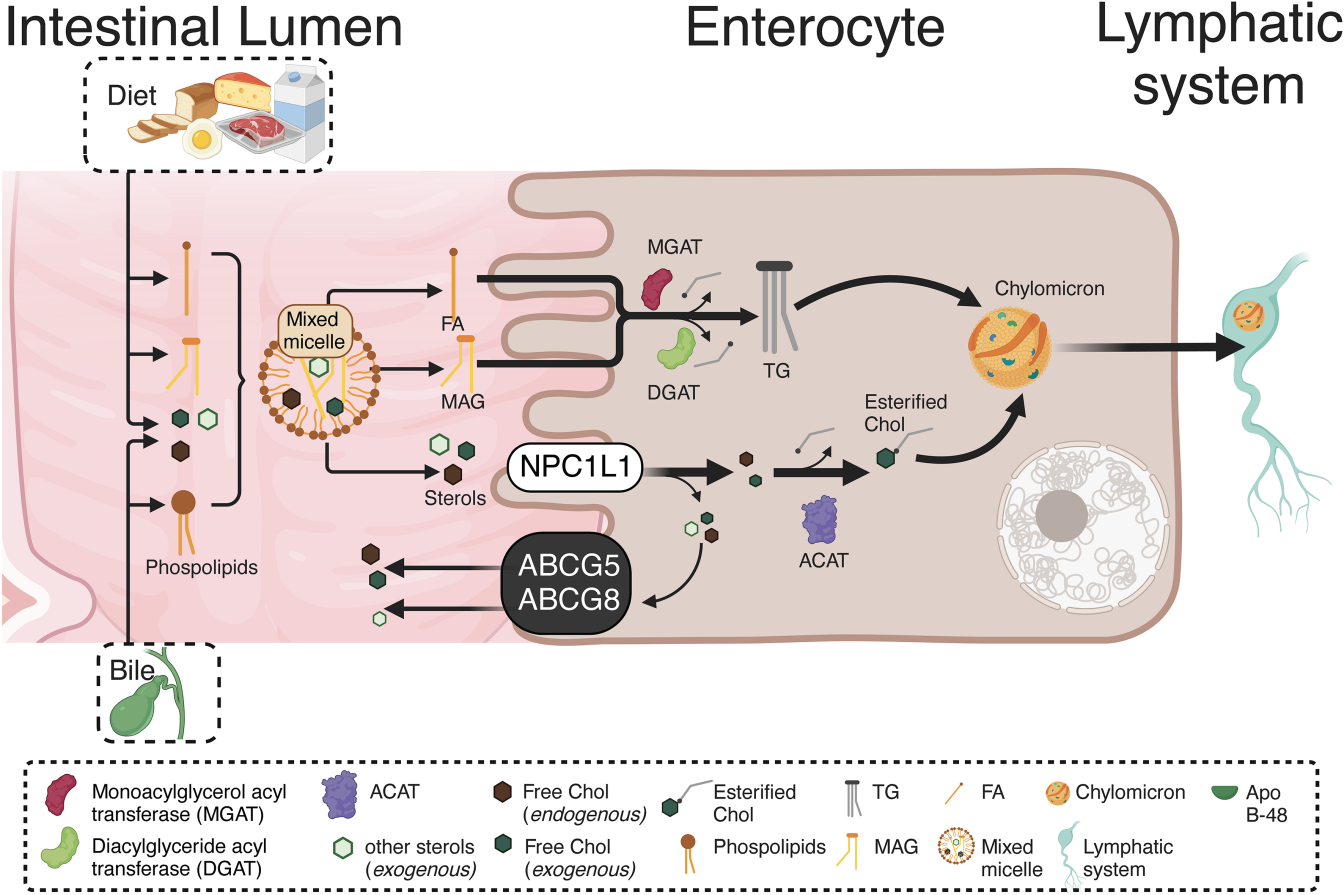
Absorption and re-esterification of exogenous and endogenous lipids and sterols by intestinal epithelial cells. Dietary cholesterol, MAGs, and free FA, inside of mixed micelles reach the enterocytes. A selective cholesterol absorption results from regulated mechanisms differentiating from other micellar lipid components. The cholesterol transport from micelles, contacting the intestinal cell microvilli, is carried out by NPCL1 through cholesterol-regulated clathrin-mediated endocytosis. Once in the enterocyte, the TG re-esterification is carried out by two enzymes: MGAT and DGAT. Both incorporate FA, the first to a MAG, while the second to a DAG, resulting in TG formation. Likewise, free cholesterol is re-esterified with FA by the ACAT. Endogenous and exogenous FA are used in re-esterification, with the TG produced by re-esterification, leaving the enterocytes for their distribution to different tissues. Therefore, in the enterocyte, re-esterified TG and cholesterol, as well as phospholipids and apo B-48, form chylomicrons. These are secreted from the basolateral cell region to the lymphatic vessels and afterward into the circulation. *MAGs, Monoacylglycerols; NPCL1, Niemann-Pick C1 Like 1; TG, Triacylglyceride; MGAT, Monoacylglycerol acyltransferase DGAT, Diacylglyceride acyltransferase; FA, Fatty acids; DAG, Diacylglycerol; ACAT, Acyl-CoA-cholesterol-acyltransferase.*. This figure was created with BioRender.com.

In the intestinal lumen, the enzyme cholesterol esterase starts cholesterol hydrolysis, resulting in both free cholesterol and esterified FA. Then, interacting with the mixed micelles and their content, dietary cholesterol, monoacylglycerols (MAGs), and free FA, reaches the enterocytes. A selective cholesterol absorption results from regulated mechanisms differentiating from other micellar lipid components. The cholesterol transport from micelles, contacting the intestinal cell microvilli, is carried out by the membrane protein Niemann-Pick C1 Like 1 (NPC1L1) through cholesterol-regulated clathrin-mediated endocytosis (37, 38). Once in the enterocyte, free cholesterol is re-esterified with FA (of endogenous or dietary origin) by the catalytic action of the enzyme acyl-CoA-cholesterol-acyltransferase (ACAT). Also, triacylglycerides (TG) from dietary fats and oils are hydrolyzed in the intestinal lumen and absorbed as free FA and MAGs, being re-esterified by the enterocytes and, in combination with cholesterol esters, phospholipids, and apoprotein B-48 (apo B-48), forming the chylomicrons. The latter are then secreted from the basolateral membrane into the lymphatic vessels and subsequently into circulation (39). **Figure 1** briefly summarizes the metabolism of endogenous and exogenous lipids and sterols by enterocytes.

The endogenous and exogenous cholesterol trafficking in cells is mediated by transporters that allow cholesterol influx or efflux. Some of these transporters are affected in IBD, causing impaired cellular cholesterol trafficking (40, 41) and abnormal accumulation of intracellular cholesterol in ER or plasma membranes. This recruits the NLRP3 and the adaptor molecule apoptosis-associated speck-like protein containing a CARD (ASC), thus promoting inflammasome oligomerization (42–44). Despite there being no information in IBD, this perturbation could contribute to the severity of the pathology.

### Cellular cholesterol dynamics in IBD

**Figure 2:**
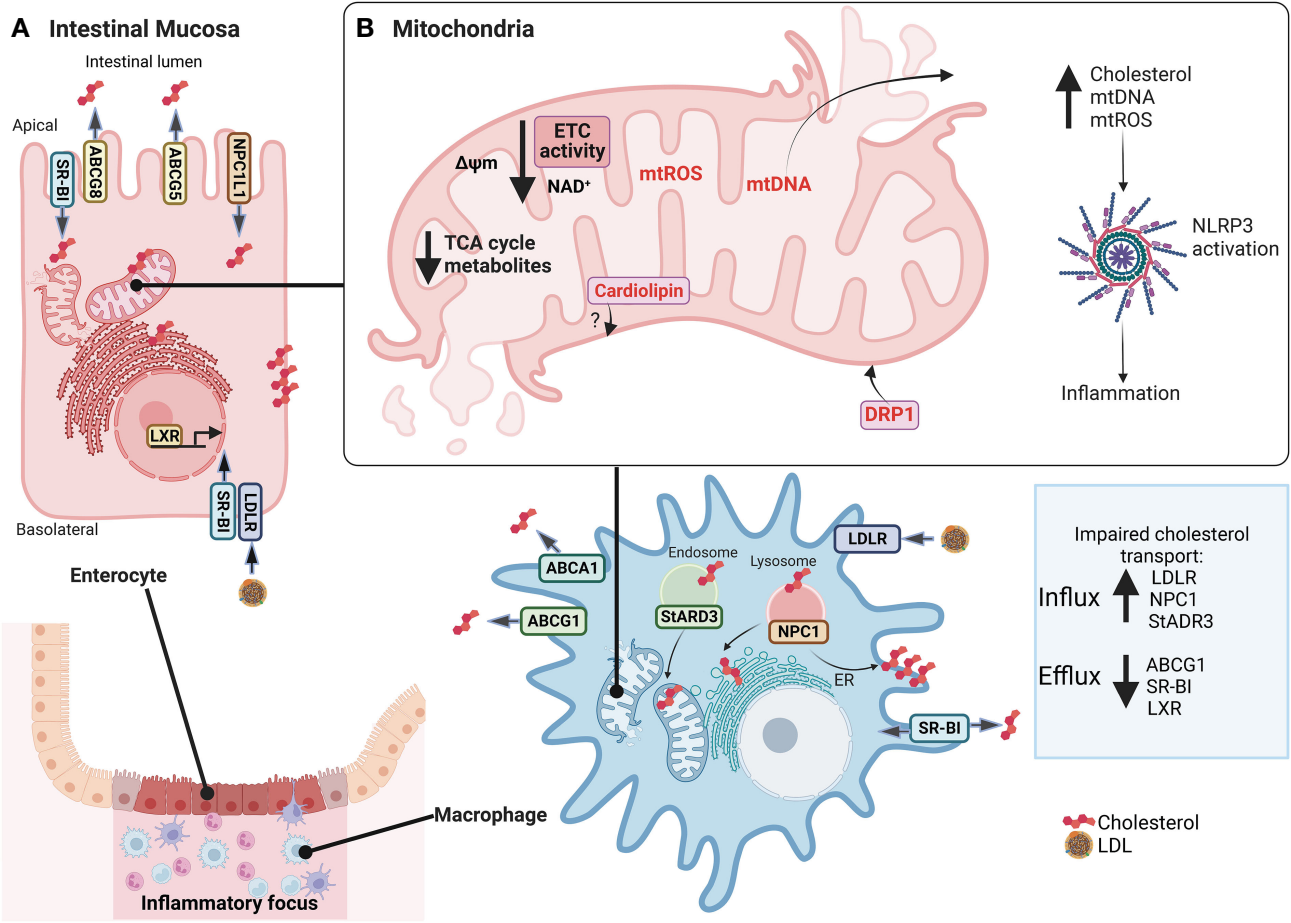
Alterations in cholesterol dynamics and mitochondrial dysfunction in IBD mucosa. In **(A)**, inflamed intestinal mucosa shows enterocytes and macrophages have impaired transport cholesterol in IBD, with increased LDL-R, NPC1, and StADR3 allowing cholesterol influx and decreased ABCG1, SR-BI, and LXR preventing cholesterol efflux. In **(B)**, also, mitochondria lose their function, promoting mitochondrial dysfunction, characterized by decreased expression of mitochondrial and nuclear genes associated with ETC and ATP synthase, NAD^+^ depletion, reduced levels of TCA cycle intermediates, reduction of Δψm, increased mtROS production, mitochondrial morphological changes with increased of DRP1 expression to regulate mitophagy and mitochondrial fission, and release of mtDNA. An imbalance in cholesterol traffic and mitochondrial function can activate NLRP3 inflammasome and promote inflammation of intestine mucosa, such as cholesterol, mtDNA, mtROS, and cardiolipin. However, there are no studies linking cardiolipin to IBD. *IBD, Inflammatory Bowel Disease; LDL-R, low-density lipoprotein receptor; NPC1, Niemann–Pick C1 protein cholesterol transporter; StADR3, Steroidogenic acute regulatory; ABCG1, ATP Binding Cassette Subfamily G Member 1; SR-BI, scavenger receptor class B type I; LXR, Liver X receptor; ETC, Electron transport chain; TCA, Tricarboxylic acid; Δψm, mitochondrial membrane potential; mtROS, mitochondrial reactive oxygen species; DRP1, dynamin-related protein 1; mtDNA, mitochondrial DNA; NLRP3, NLR (Nod-like receptor) family pyrin domain containing 3*. This figure was created with BioRender.com.

Cellular lipid fate starts with intestinal absorption of endogenous and exogenous sources to the lymphatic circulation, then transported to the liver, and finally to the bloodstream by LDL lipoproteins. Selective cellular LDL and HDL lipoprotein uptake by LDL receptors comprise a route through classical-clathrin coat endosomal/lysosomal route (45) and scavenger receptor class B type 1 (SR-BI), respectively (46). Intracellular cholesterol excess is delivered to HDL (*reverse cholesterol transport*) by ATP-Binding Cassette Transporter A1 (ABCA1), ATP-binding cassette transporter G1 (ABCG1), and SR-BI (47). Additionally, other transporters are steroidogenic acute regulatory (StAR) family members, including StARD1 and StARD3, mediating cholesterol movement from endosomes to mitochondria and ER (48, 49). Furthermore, vesicular transporter NPC1 mediates cholesterol efflux from late endosomes/lysosomes to the ER and plasma membranes (50). As expected, NPC1 deficiency in Niemann Pick C disease (NPC) is characterized by cholesterol accumulation in lysosomes and altered cholesterol content in mitochondria, plasma membranes, and ER (51), whereas NPC1 overexpression in CHO cells increases plasma membrane and ER cholesterol content (52). Notably, *Npc1*-null mice show impaired autophagy, intestinal granuloma, and a CD phenotype (53). Likewise, CD patients with an NPC1 disorder develop a granulomatous aggressive phenotype (54). Interestingly, IBD patients show increased *LDL-R*, *NPC1*, and decreased *ABCG1* and *SR-BI* transcript levels (40, 41) suggesting that cellular cholesterol dynamics and homeostasis are affected in the intestinal mucosa **(** **Figure 2** **)**.

Also involved in cholesterol homeostasis and TG metabolism in the intestinal mucosa are the nuclear receptors (NR), liver X receptor (LXRs) α (*NR1H3*) and β (*NR1H2*) (55), as evidenced by decreased LXR expression in IBD patients, and several colitis mouse models (*Il-10*-null mice and dextran sodium sulfate, DSS or 2,4,6-trinitrobenzene sulfonic acid, TNBS-induced colitis) (56, 57) accompanied by decreased expression of *ABCA1* (an LXR target gene) (57).

Interestingly, IBD etiology is associated with an unbalanced peripheral lipid profile with low total cholesterol, HDL, and LDL (58, 59). Furthermore, disease severity is related to high TG levels (59) and hyperlipidemia in IBD patients, and colitis mouse models (60, 61) suggesting the strong linkage between lipid metabolism and IBD. Moreover, a recent study demonstrates that the visceral adipose tissue is associated with therapy response and lower remission rates in IBD patients (62). In addition, the use of statins (drugs lowering circulating cholesterol levels) in IBD is associated with reduced steroids use (63) and disease relapse risk.

Being a component of plasma membranes and endomembranes, cholesterol is a critical constituent in lipid rafts. Cholesterol is decreased in lipid rafts and increased in non-lipid rafts fraction in an *in vitro* DSS-induced IBD-like phenotype of intestinal Caco-2 cells, with ER showing altered trafficking of protein, glycoprotein, and cholesterol. All this suggests inflammation promotes both unbalanced cell polarity and membrane integrity (64). In line, macromolecule trafficking is also disturbed by ER stress in IBD, characterized by increased expression of ER stress proteins (immunoglobulin-binding protein (BiP), C/EBP homologous protein (CHOP), transcription factor activation protein 4 (ATF4), and X-box binding protein (XBP1)) (64, 65). Mitochondria-associated ER membranes (MAMs) are microdomains that allow the calcium and lipid trafficking between mitochondria and ER, although disturbed regulation contributes to pathological conditions (66). Under these ER stress conditions, interruption of this ER-mitochondria communication produces oxidative stress and mitochondrial depolarization, impairing mitochondrial bioenergetics. A pro-inflammatory *in vitro* model of the M1-like Mø phenotype exhibits reduced ER-mitochondria communication and upregulated genes involved in mitochondrial fusion (*MFN1*, *MFN2*, and *OPA1*) and fission (*DRP1* and *FIS1*), compared to M0- and M2-like Mø. Additionally, M1-like Mø converts superoxide anions into hydrogen peroxide (H_2_O_2_), generating mitochondrial ROS (mtROS) release (67, 68). Although changes in ER-mitochondria communication in cells from inflamed mucosa, such as Mø in IBD, are so far unknown, *in vitro* M1-like Mø models suggest alterations in the reciprocity platform between organelles (67).

Concomitantly, mucosal StARD3 expression is also increased in IBD patients (69), suggesting an increased cholesterol content in mitochondria. Accordingly, StARD3 upregulation increases mitochondrial cholesterol content in mice on a cholesterol-rich diet (70). Similarly, hepatocytes overexpressing StARD3 cause mitochondrial dysfunction mediated by oxidative stress, comparable to *Npc1*-null mice suffering severe mitochondrial oxidative stress (71). Currently, no studies demonstrate cholesterol levels within the mitochondrial membrane in the intestinal cell types in IBD patients or IBD *in vivo* models. On the other hand, cholesterol sulfate, which is catalyzed by hydroxysteroid sulfotransferase 2B1 (SULT2B1), and intestinal epithelium-specific knock of *Sult2b1* has been significantly associated with down-regulation of cholesterol biosynthesis genes. In addition, cholesterol sulfate administration improved colitis features in DSS mice model; however, further studies are necessary to evaluate its anti-inflammatory effect in IBD (72).

## Impaired mitochondrial metabolism in IBD

Mitochondrial dysfunction refers to loss of electron transport chain (ETC) efficiency, reduced ATP synthesis, mitochondrial morphological changes, and increased mtROS, and these are present in chronic diseases (58) such as IBD **(** **Figure 2** **)** (73–76). Regarding IBD, decreased expression of some mitochondrial and nuclear genes, associated with ETC (e.g. Complex I, III, and IV), ATP synthase (Complex V), and tricarboxylic acid (TCA) cycle, as well as reduced ETC activity has been observed in inflamed colonic biopsies versus non-inflamed mucosa (73). Despite the above, conflicting evidence on the contribution of ETC to IBD suggests a possibly high intrinsic variability in patients. Whether this is related to the severity of the pathology or the response to therapy currently remains unclear.

Complex I activity is a limiting step in oxidative phosphorylation (OXPHOS) and catalyzes the oxidation of nicotinamide adenine dinucleotide (NADH) (77, 78). Simultaneously, complex II links ETC with TCA cycle, oxidizing succinate to fumarate and reducing flavin adenine dinucleotide (FAD^+^) to hydroquinone form (FADH) (79). In UC patients, NAD^+^ is depleted in inflamed tissue (80). Although no FAD^+^ data is available in IBD, lower levels are expected due to decreased complex II activity; however, further studies are necessary to verify this.

Consistent with this reduced respiratory complex activity, reduced levels of TCA cycle intermediates citric acid, fumarate, isocitric acid, malate, pyruvate, and succinate have been reported in inflamed mucosa of UC patients (81). Seemingly even more significant, citrate, aconitate, α-ketoglutarate, succinate, fumarate, and malate are decreased in CD compared to UC and healthy patients (82), suggesting a differential metabolic phenotype related to bioenergetics dysfunction and inflammation in CD.

Notably, a metabolic shift towards glycolysis to compensate for a deficiency in mitochondrial ATP synthesis is shown in PBMCs from IBD patients when compared to healthy donors (83). In this IBD metabolic context, colonocytes change to glycolytic metabolism (increased intracellular lactate), and bacteria in the intestinal lumen consume epithelium oxygen, causing dysbiosis and promoting mucosal inflammation (84).

Colonic epithelial cells present a reduction of mitochondrial membrane potential (Δψm) in inflamed tissue (73) that could be promoted by the reduction of ETC activity and the presence of uncoupling proteins (UCP), which permit the proton leak from the mitochondrial intermembrane space to the matrix (85). *Ucp2* overexpression was reported in colon of DSS-treated mice (86), with a reduced inflammation severity in the *Ucp2* knockdown (87) that could promote the loss of Δψm. However, discordant results were obtained in DSS-treated *Ucp2* null mice, which developed more severe colitis that was associated with higher ROS and decreased expression of ZO-1 and JAM1 (88) (related to tight junction permeability and epithelial architecture loss). Furthermore, *Ucp2* expression in colonocytes in the small intestine from TNBS-treated mice was not significantly increased, despite the reduction of respiration in isolated mitochondria (89) possibly reflecting the participation of other proteins of the mitochondrial membrane. More studies are necessary to evaluate the participation of UCP2 in IBD bioenergetics and physiopathology.

Regarding mitochondrial morphology, CD patients with active disease show severe ultrastructural mitochondrial abnormalities in Paneth cells, goblet cells, and enterocytes (90). Intestinal enterocyte mitochondria appear dysmorphic, with cristae destruction and abnormal architecture in mouse models of intestinal inflammation. Dysfunctional mitochondria undergo BCL2 interacting protein 3-like (BNIP3L)/NIX-mediated mitophagy and mitochondrial fission, evidenced by increased dynamin-related protein 1 (DRP1) expression. As a physiological response to stress and mitochondrial damage, hypoxia-inducible factor alpha (HIF1α) and mtROS are enhanced to regulate mitophagy during intestinal inflammation, eliminating dysfunctional mitochondria from the cell and preventing further ROS accumulation (91).

Mitochondrial dysfunction in IBD has also been related to decreased *PPARGC1A* expression (encoding Peroxisome proliferator- activated receptor-alpha; PGC1-α), which is considered a master regulator of mitochondrial biogenesis and bioenergetics (69, 73). Under stress conditions, PGC-1α potently stimulates mitochondrial turnover and antioxidant activity (92, 93). Different roles for PGC-1α have been suggested from *in vitro* and *in vivo* UC models. PGC-1α is highly expressed in the murine intestinal epithelium; however, during colitis, acetylation inactivates PGC-1α, followed by ubiquitination and proteasomal degradation (92). Moreover, hypermethylation of the *PPARGC1A* gene in severe UC, when compared to mild UC patients, is related to decreased PGC-1α expression (73, 94). In addition, reduced mitochondrial mass in the intestinal mucosa of UC patients can be a consequence of decreased PGC-1α and/or increased mitophagy (95). Epithelium-specific *Ppargc1a* null mice are more susceptible to DSS-induced colitis compared to wild-type mice (92), highlighting its participation in colitis pathophysiology. Contradictorily, Paneth cells of CD patients showed upregulated PGC-1α signaling, which may represent an adaptive response to ongoing mitochondrial damage during IBD (90).

Post-translational regulation of PGC-1α involves highly conserved NAD^+^-dependent deacetylase Sirtuins (SIRTs), including SIRT1. SIRT1 senses metabolic stress and thus promotes upregulation of mitochondrial function and FA oxidation genes (96, 97). Also, SIRT1 deacetylates histones (H1, H3, and H4) and transcription factors, such as NF-κBp65, inhibiting pro-inflammatory gene transcription. Accordingly, decreased SIRT1 in IBD mucosa promotes the inflammatory environment (98).

Conventional therapy in IBD, such as 5-ASA (5-aminosalicylic acid), reverses mitochondrial dysfunction by activating Peroxisome proliferator- activated receptor-gamma (PPAR-γ) (84, 99, 100). PPAR-α and PPAR-γ nuclear receptors (encoded by *PPARA* and *PPARG)*, expressed in crypt upper epithelial cells and Mø, are associated with cellular processes, such as mitochondrial biogenesis and translation initiation. Interestingly, PPAR-α and PPAR-γ have been associated with ROS detoxification and cholesterol biosynthesis, while specifically PPAR-γ is associated with TCA cycle and NF-κB signaling inhibition, whose gene expression is decreased in IBD, affecting these processes (73, 101). Overexpression of PPAR-γ (in a hepatocyte cell line) decreases *HMGCR* and *SREBP-*2 gene and protein levels, increasing *CYP7A1*, *ABCG5*, and *ABCG8* gene and protein expression, which contribute to a decrease in cholesterol synthesis, an increased conversion of cholesterol to BAs and cholesterol efflux (102).

In mouse Mø treated with soluble (free) cholesterol, it was observed that cholesterol was absorbed and accumulated in the cytoplasm and mitochondria, leading to increased mitochondrial mass, decreased oxygen consumption rate, and OXPHOS (103). This suggests that an accumulation of cholesterol in cellular compartments, such as the mitochondria, could cause changes in mitochondrial metabolism and bioenergetics. Further studies of intracellular cholesterol accumulation and trafficking with its metabolic consequences in IBD are still required.

## Inflammasome involvement in IBD

### Inflammasome activation

Participating in innate antimicrobial responses against PAMPs, DAMPs, and stress, the inflammasome is a supramolecular complex composed of a sensor protein, a caspase, and an ASC adapter protein (104). Sensor proteins are PRRs of innate immunity, belonging to the NOD-like receptor family (NLR), such as NLRP1, NLRP3/NLRP6, NLRC4, and NAIP, or the PYHIN family (pyrin and HIN200 (hematopoietic interferon-inducible nuclear antigens with 200 amino acid repeat domain-containing protein)), such as AIM2/IFI16 (105, 106). So far, NLRP3 is the type most studied. Inactive NLRP3 resides mainly in the ER but, upon activation, translocates to MAMs in the perinuclear space and recruits ASC and caspase-1, thereby enabling cytoplasmic NLRP3 inflammasome assembly (107). In modulating NLRP3 activation, its filamentous assembly is ASC-dependent, containing a central nucleotide-binding, self-oligomerization domain (NBD) with ATPase activity and an LRR (leucine-rich repeat) domain conserved at the C-terminal. Once activated, NLRP3 oligomerizes, assembling multiple ASC filaments, through interactions between both N-terminal PYD (pyrin domain) of NLRP3 and ASC. Alternatively, ASC contains a caspase activation and recruiting domain (CARD), which interacts with the caspase-1 CARD, linking caspase domains for dimerization, activating self-cleavage, and finally, caspase-1 activation (108).

The canonical inflammasome pathway activation involves caspase-1-mediated cleavage of pro-IL-1β and pro-IL-18 into active IL-1β and IL-18 (104). Moreover, the non-canonical pathway consists of ASC-independent murine caspase 11 or human caspase 4/5, NRLP3 (109). Caspase-1 and caspase-4/5/11 cleave gasdermin D (GSDMD), releasing gasdermin-amino terminal domain and inducing pyroptosis, a variant of programmed cell death characterized by pore formation in the plasma membranes. This allows cytoplasmic content to be released from infected cells, which are then eliminated by neutrophils (110).

As mentioned before, inflammasome activation requires two signals, one being NF-κB-mediated *NLRP3* and pro-*IL1B* gene transcription, and a second signal may arise from PAMPs, such as LPS, toxins, viral and bacterial nucleic acids and leads to oligomerization and activation. In addition, the inflammasome senses stress and cell damage, such as high ATP concentrations, mitochondrial-derived components (cardiolipin, ROS, mitochondrial DNA (mtDNA)), ER stress, intracellular calcium influx, uric acid, serum amyloid A, and cholesterol crystals (20–23).

### Inflammasome activation in IBD

Inflammasome activation in the intestinal mucosa promotes pathogen clearance and Mø-mediated epithelial barrier recovery, with NLRP3 expressed both in colonic epithelial cells and Mø (111). The inflammasome is linked to IBD, as higher *NLRP3*, *Caspase-1*, NIMA-related kinase 7 (*NEK7*), and *GSDMD* expression were observed in inflamed versus non-inflamed tissue of UC patients (18). Also, hypomethylation of genes, such as *NLRP3, NLRC4, NLRP12*, and *IL-10*, correlated with higher gene transcription in severe compared to mild UC patients (94).

Furthermore, intestinal barrier dysfunction in DSS-induced colitis in mice allows the translocation of bacteria to stimulate lamina propria Mø *via* TLR/NF-κB, leading to pro-IL-1β and pro-IL-18 transcription, consequently NLRP3 activation (18, 112). This observation was confirmed by DSS-treated Mø *in vitro*, producing high IL-1β levels in a caspase-1-, NLRP3- and ASC-dependent manner (29). Nonetheless, conflicting evidence on the role of *Nlrp3* and *Caspase 1* genes was observed in IBD models, reporting in *nlrp3*-null mice less severe colitis and lower colonic pro-inflammatory cytokine production (29, 112). Whilst another study showed worse survival and clinical scores than wild-type mice (113). Recently, a GWAS study demonstrated the presence of inflammasome activation components in an IBD risk loci (33).

Additionally, the inflammasome is essential for protection against colitis-associated dysplasia and tumorigenesis towards colorectal cancer (CRC) (31, 113). Both *Nlrp3-* and *Caspase 1*-null mice treated with AOM (azoxymethane)/DSS develop large tumors with significant hyperplasia, and inflammation, with reduced production of IL-18 and the presence of proliferating dysplastic cells in dysplasia regions. NLRP3-dependent IL-18 production prevents neoplasia, with IL-18 participating in intestinal epithelial barrier repair *via* IFN-γ production and STAT1 signaling (31). In data analysis on the Oncomine^®^ platform from patients with CRC, a decreased expression of inflammasome *NLRP1, NLRP3, NLRC4*, and *AIM2* was observed compared to healthy controls (114).

Thus, evidence from *in vivo* colitis models shows that other molecular factors could interact with the inflammasome.

### Cholesterol balance and inflammasome activation

Altered cholesterol transport leads to its accumulation in ER or plasma membranes, recruiting NLRP3 and ASC, facilitating inflammasome oligomerization (42–44), thus, impacting inflammation in atherosclerosis and Alzheimer’s disease (115). Initially, cholesterol uptake in intestinal epithelial cells by NPC1L1 promotes acute intestinal inflammation, recruiting myeloid cells, such as Mø, which capture cholesterol by NPC1 and activate NLRP3 inflammasome by activating caspase-1 and IL-1β secretion (42, 116). Additionally, dietary cholesterol has been shown to exacerbate CRC in murine models by activating NLRP3 inflammasome (20), and its blockade improves metabolism in obese mice (117). Additionally, cholesterol accumulation in plasma membranes (rafts/caveolae domains) affects its integrity, mainly represented by a changed distribution of caveolin-1 (CAV-1), becoming a platform for the inflammasome (118), and TLR4 activation in immune cells (44, 119–122). Importantly, TLR4 monomer is inactive in the plasma membranes and recruited into lipid raft domains once stimulated, allowing the toll-interleukin-1 receptor (TIR) domain to interact with membrane-associated co-receptor MAL/MyD88, initiating signaling for pro-IL-1β production (123). Reinforcing the role of cholesterol transport in inflammasome activation, ABCA1/G1 deficiency promotes cholesterol accumulation in Mø (43, 124, 125). Similarly, treatment with the NPC1 inhibitor U18666A (126) or NPC1 deficiency leads to the cholesterol accumulation in late-endosomes/lysosomes, reducing cholesterol transport to the ER and plasma membranes, thus blocking inflammasome assembly (42). These observations demonstrate a role for NPC1 in cholesterol homeostasis and TLR4 activation impacting inflammasome assembly. However, these mechanisms have not been established yet in IBD.

### Inflammasome activation by mitochondrial mediators in IBD

The NLRP3 inflammasome can be activated by mitochondrial DAMPs which are released from intestinal mucosa during mitochondrial dysfunction in IBD (127). Mitochondrial damage causes mtDNA fragmentation and mtROS production, which oxidizes mtDNA into an oxidized form (ox-mtDNA) and acts as a second signal to activate the NLRP3 inflammasome (128). On the other hand, the first signal in LPS-stimulated Mø induces *de novo* mtDNA synthesis *via* the MyD88/TRIF-dependent TLR4, which activates IRF1 to induce CMPK2 expression, a mitochondrial nucleotide kinase (129). This *de novo* mtDNA is susceptible to oxidation and fragmentation, resulting in ox-mtDNA fragments, which can activate the inflammasome (129). Also, NLRP3 inflammasome activation in Mø induces pyroptosis by GSDMD. This process is characterized by plasma membrane permeabilization, causing changes in ion homeostasis that contribute to mitochondrial collapse, thus accumulating cytosolic mtDNA to be released to the extracellular environment when the cell undergoes apoptosis or pyroptosis (130). Both IBD patients with active disease and DSS-induced colitis in mice show increased plasma mtDNA levels, corroborating mitochondrial damage and suggesting inflammasome activation by mtDNA during IBD development (127).

NLRP3 inflammasome can also be activated by direct interaction with mitochondrial cardiolipin, in a mtROS-independent manner (21). Cardiolipin is a phospholipid locates exclusively in the inner mitochondrial membrane (IMM). It can translocate to the outer mitochondrial membrane (OMM) by mtROS production, PAMPs (e.g., LPS), or pro-apoptotic stimuli, thus promoting mitophagy (131). This suggests that cardiolipin could have a role in IBD pathogenesis. However, there are still no studies linking cardiolipin to IBD.

## The interplay between mitochondrial dysfunction, cholesterol metabolism, and inflammasome activation upon the gut microbiota in IBD

Important host’s physiological functions are influenced by their gut microbiota, whose closest association became a single unit, called “holobiont”, where mtROS levels have important roles. Because of mtDNA mutations, mtROS levels are increased, reducing the ATP production, with MtDNA variations have an important impact on intestinal health. Mice strains with increased OXPHOS are less susceptible to developing colitis (PMID: 23872498), while mtDNA variations lead to different mtROS levels, influencing gut microbiota (132, 133). Furthermore, mice with mtDNA variants related to higher ROS production had a reduced gut microbiome diversity (133). In IBD, there is reduced microbial diversity, lower SCFAs and ATP production, impaired mucus production and antimicrobial peptides (134, 135), as a consequence of higher pathogenic bacteria and mtROS levels. IBD patients present different microbiota compositions; some having eubiotic (homeostatic) or dysbiotic (imbalanced) microbiota. Increased facultative anaerobes and aerobic bacteria is seen in dysbiotic IBD patients compared to healthy control and eubiotic IBD patients (characterized by high obligate anaerobe abundance) (136). This evidence is consistent with the hemoglobin (oxygen carrier) and ROS release to the intestinal lumen, increasing oxygen levels to overgrown facultative anaerobes and aerobic bacteria (137). This dysbiotic environment promotes increased gut permeability, thus favoring the passage of PAMPs, which can activate PRRs, such as the TLR4/NLRP3 inflammasome signaling pathway, and triggering inflammation.

Disruptions in cholesterol metabolism are described in IBD, where BAs are end products of cholesterol, and their synthesis is regulated by the farnesoid X receptor (FXR). In IBD, there is a decreasing activity of FXR (138), a consequence of exacerbated NF-κB activation (139, 140).

Despite the pathogenic role of BAs in IBD being controversial, an impaired reabsorption of conjugated BAs occurs in enterocytes, and consequently, many side effects such as maldigestion of lipids, steatorrhea, impaired intestinal motility, and dysbiosis can occur (141). Regarding bile acid malabsorption, a parallel stimulation of cholesterol synthesis, cholesterol degradation, and LDL receptor expression, results in reduced LDL cholesterol in IBD patients (142). On the other hand, in humans, conjugated BAs are secreted in the intestine, being substrates of bacterial microbiota, resulting in the production of secondary BAs, such as deoxycholic and lithocholic acids (DCA and LCA, respectively) (143). These can activate TGR5 and induce the TGR5-cAMP-PKA signaling pathway, promoting NLRP3 ubiquitination (144). Accordingly, several studies demonstrate reduced DCA and LCA levels in active IBD patients, associated with gut dysbiosis (143), and consequently, NLRP3 can be permanently activated, exacerbating the inflammatory response.

## Dietary phytochemicals to restore mitochondrial function and cholesterol balance in IBD

**Figure 3:**
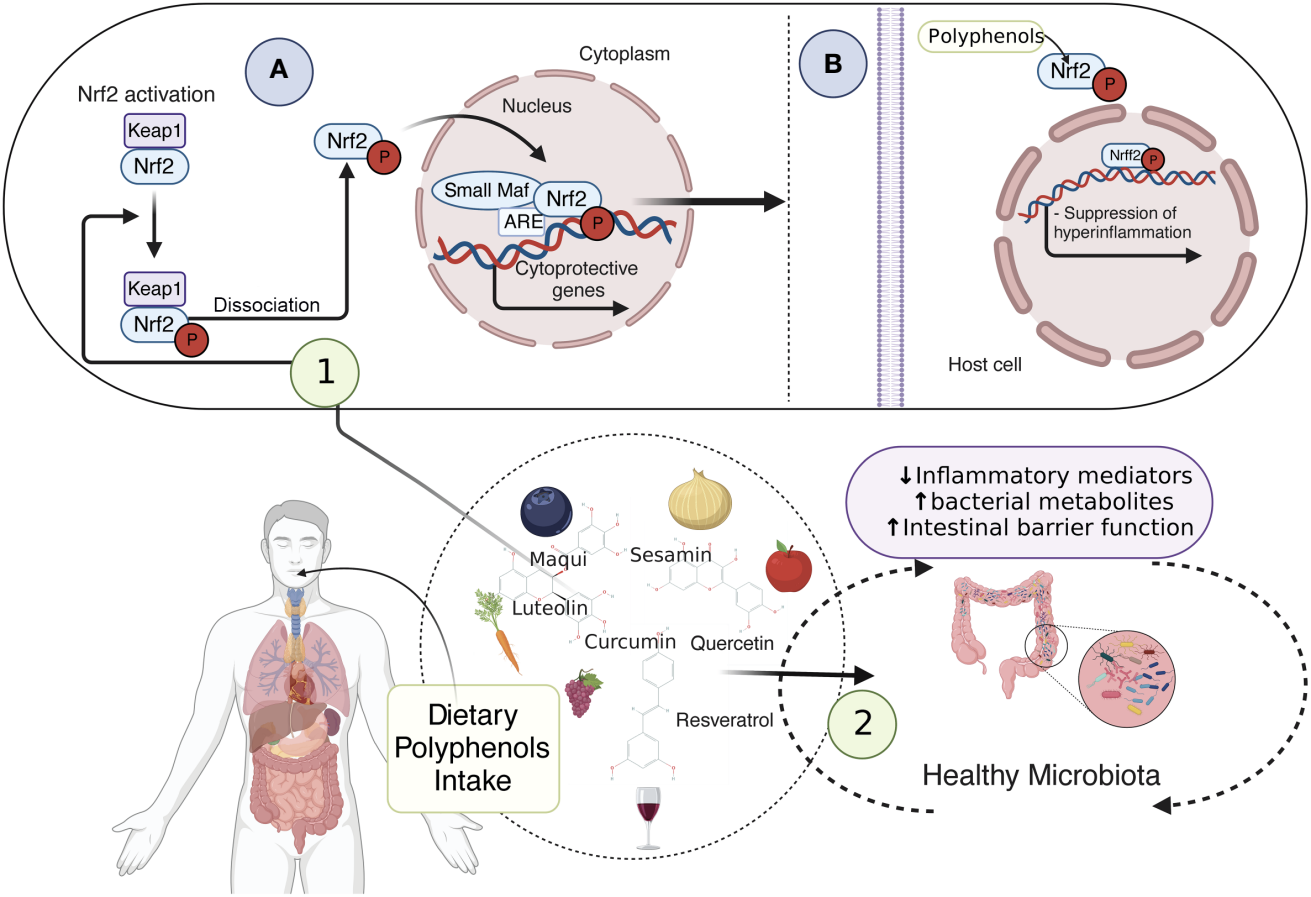
Dietary compounds and activation of Nrf2 pathway preventing inflammation. dietary compounds, such as polyphenols, prevent oxidative stress, mitochondrial dysfunction, and intracellular cholesterol trafficking *via* Nrf2 signaling. Sequestered in the cytoplasm by Keap1, Nrf2 is inactive, however, when phosphorylated by polyphenols, it can be released from Keap1, translocate to the nucleus, and form a heterodimer with small Maf proteins. This allows cellular protection and adaptive responses through the cytoprotective gene expression (1**A**), thus suppressing the inflammation (1**B**). Additionally, phytochemicals acting as prebiotics promote a healthy microbiota, which ensures an accurate amount of bacterial metabolites, enhancing the epithelial barrier function and reducing the inflammatory response (2). *Nrf2, Nuclear erythroid 2-related factor 2; Keap1, Kelch-like ECH-associated protein1*. This figure was created with BioRender.com.

**Figure 4:**
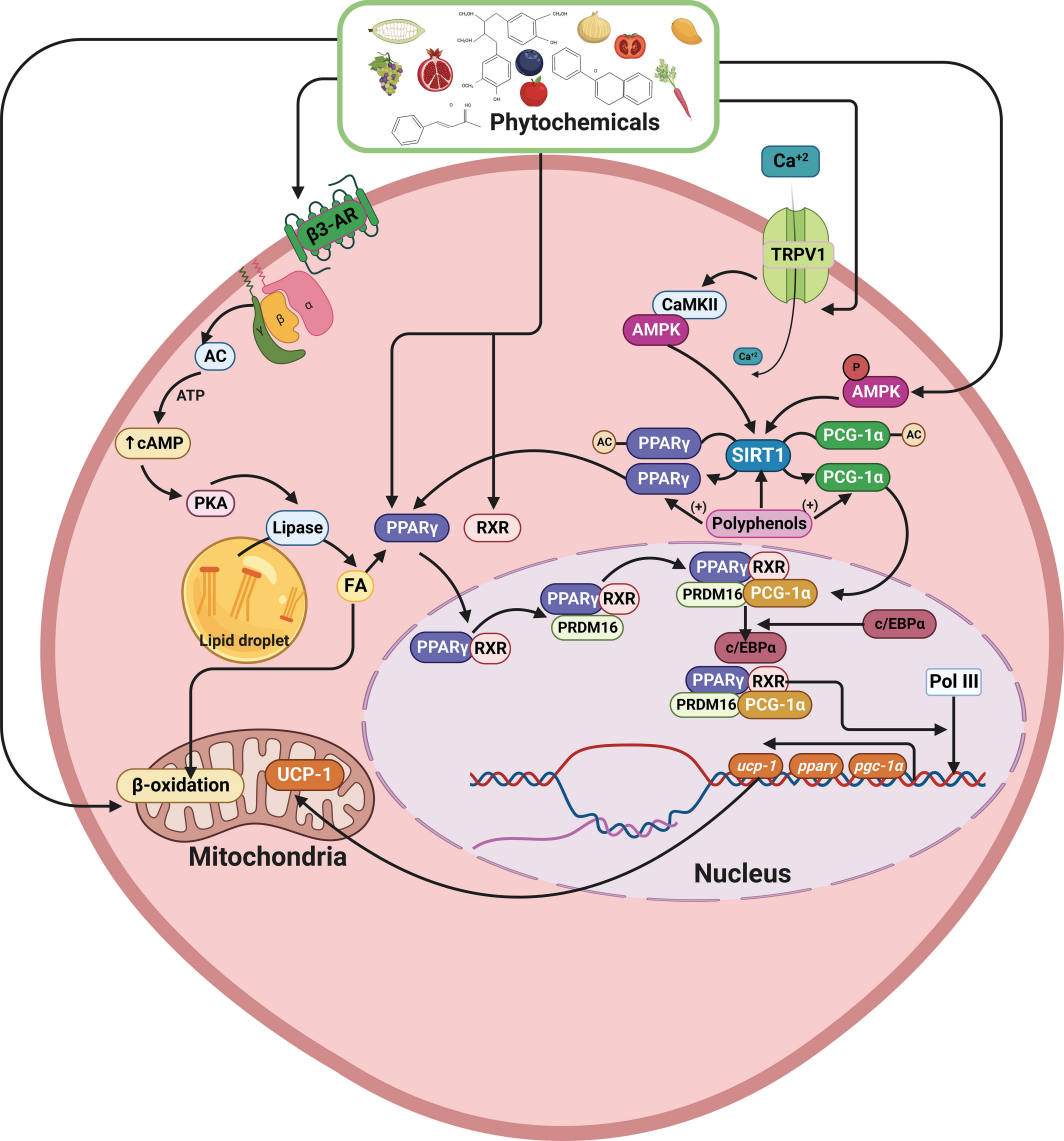
Schematic model showing the effects of phytochemicals on lipid metabolism, increase of PPAR-γ SIRT1, PGC-1α, and UCP1 expression. Polyphenols can deacetylate PPAR-γ and PRDM16, promoting the UCP1, PPAR-γ, SIRT1, and PGC-1α overexpression. On the other hand, *via* TRPV1, polyphenols can increase SIRT1 expression, and through β3-AR activation, increase cAMP levels, activating MAPKs pathway, resulting in TG hydrolysis, FA oxidation, and increased mitochondrial UCP1 transcription and activity. *PPAR-γ, Peroxisome proliferator- activated receptor-gamma; SIRT1, Sirtuin 1; PGC-1α, Peroxisome proliferator- activated receptor-alpha; PRDM16, PR domaing containing 16; UCP1, Uncoupling protein 1; TRPV1, Transient receptor potential vanilloid 1; β3-AR, β3-adrenergic receptor; TG, Triacylglyceride; FA, Fatty acid*. This figure was created with BioRender.com.

Since mtROS overproduction and cholesterol unbalance are considered detrimental events in IBD, novel antioxidant strategies targeting the mitochondrial metabolism may be promising alternatives and/or supportive therapies (90, 145, 146). Notably, dietary compounds (mainly phytochemicals, such as polyphenols) may counteract oxidative stress, mitochondrial dysfunction, and intracellular cholesterol trafficking *via* Nuclear erythroid 2-related factor 2 (Nrf2) (**Figure 3**), PPAR-γ (**Figure 4**) and LXR signaling. These effects have been extensively explored and reviewed elsewhere. However, the main findings relevant to this review regarding phytochemicals are summarized in **Table 1**. In addition, phytochemicals can act as prebiotics, promoting an eubiotic microbiota that produces higher amounts of bacterial metabolites, enhancing the epithelial barrier function, and reducing the inflammatory response, which could be related to the improvement of symptoms in IBD patients (**Figure 3**) (1, 174).

**Table 1:** Dietary phytochemicals and their restorative effects on mitochondrial function and lipid homeostasis.

Phytochemical (Source)	Outcome	Proposed pathway	Model	Reference
Resveratrol (Grape, wine, peanut, and cranberry)	Anti-inflammatory protection and oxidative stress inhibition against intestinal inflammation.	Nrf2 activation: ↑Oxygenase-1 (HO-1) mRNA, ↓ROS production, and ↑PPAR-γ accumulation.	cytokine-stimulated (IL-1α, TNF-α, IFN-γ) HT-29 cells	(147)
	Anti-inflammatory effects.	↑Nrf2, ↓IL-1β, and ↑IL-11.	*In vitro* UC model in Caco-2 cells challenged with TNF-α.	(148)
	Protective effects against the alterations of mitochondrial function and oxidative stress.	↑Intracellular ATP, protective effects against ↓Δψm induced by INDO.	Intestinal epithelial Caco-2 cells induced by indomethacin (INDO).	(149)
	↓Disease activity and ↑Quality of life in UC patients at least partially through the ↓Oxidative stress.	–	Prospective, randomized, double-blind, placebo-controlled study in UC patients. Supplements (containing 500 mg trans-resveratrol) or placebo capsules.	(150)
	Anti-atherosclerotic effects: ↓FA and MAG intestinal accumulation. Restoration of succinate and lactic acid levels.	Abolishes oleate-triggered lipid, total cholesterol, and esterified cholesterol accumulation by activating PPAR-α and PPAR-γ signaling.	*ApoE* null mice fed with a high-fat diet (HFD) (AS) and resveratrol intervention. RAW 264.7 mice Mø treated with oleate and resveratrol.	(151)
Quercetin (onion, apple grape, and citrus fruits)	Protective effects against the alterations of mitochondrial function and oxidative stress.	↑Intracellular ATP, protective effects against ↓Δψm induced by INDO, and inhibition of the inhibitory effects of INDO and rotenone on complex I.	Intestinal epithelial Caco-2 cells induced by INDO.	(149)
	Mitochondrial protective effects against and maintenance of gastrointestinal mucosal renewing regulating apoptosis.	Prevents Ca^2+^ mobilization induced by INDO and its consequences, including ↑Caspase-3 and caspase-9 activation and cytochrome C release.	Intestinal epithelial Caco-2 cells induced by INDO.	(152)
	↓NLRP3 inflammasome activation and ↓Mitochondrial damage.	↓Activity of caspase-1 and ↓Secretion of IL-1β and ↓IL-18 *via* NLRP3 inflammasome. Improvement in Δψm, blocking cytochrome C release, ↓O_2_ consumption, ↓ mtDNA cytosolic content, and ↓ ROS level.	Caco-2 cells infected by *Escherichia coli* O157:H7	(153)
	Intestinal anti-inflammatory effects *via* Nrf2/HO-1	↓TNF-α, IFN-γ, and IL-6. Nuclear Nrf2 accumulation ↑ HO-1 expression in colonic Mø.	T cell-dependent colitis model induced by the adoptive transfer of *naive* T cells into *Rag1* null mice and DSS-induced colitis mice model.	(154)
Sulforaphane (cruciferous vegetables)	Antioxidant and anti-inflammatory effects, ↑ Mitochondrial bioenergetic function upon cholesterol-induced pancreatic β-cell dysfunction.	Improving ATP turnover, spare capacity, and impairment of the electron flow at complexes I, II, and IV. ↓NFκB pathway.	Min6 cells, a β-cell line exposed to high concentration of cholesterol.	(155)
	↓Intestinal permeability upon LPS, ↓Oxidative stress, ↓Inflammation, and ↓apoptosis.	↑SIRT1 and ↑PGC-1α expression. ↑ Antioxidant enzymes of the Nrf2 pathway and ↓Lipid peroxidation induced by cholesterol. Activating the AMPK/SIRT1/PGC-1α pathway.	LPS-induced Caco-2 *in vitro* model.	(156)
Dried apple peel polyphenols (DAPP)	↓DSS-induced damage, ↓Pro-inflammatory factors, ↓Oxidative markers, and ↓ROS. ↓Mitochondria-dependent cell death, ↑β-oxidation, ↑Mitochondrial bioenergetics, and ↓Alteration in mitochondrial morphology.	↓TNF-α, COX-2, and iNOS.	*In vivo* model of DSS-induced colitis in male C57BL6 mice.	(157)
Strawberry Ellagitannin-Rich Extract (S-ET)	↓ HFD effects in rats, ↓Body weight, ↓Relative mass of the epididymal pad, ↓Hepatic fat, ↓Oxidized glutathione, ↓TG, ↓Total cholesterol, and ↓Thiobarbituric acid-reactive substances concentrations and improve blood plasma parameters.	↓H_2_O_2_ and SOD2 protein expression and ↑8-oxoguanine DNA glycosylase 1 (OGG1) expression. ↑CPT-1 and ACADL, ATP production, and PGC-1α. ↓NF-kB and AP-1.	HFD supplemented with S-ET) in Male Wistar rats. *In vivo* model	(158)
Luteolin (carrot, pepper, celery, spinach, and parsley)	Antioxidants and anti-inflammatory effects.	↑ Nrf2 and ↓iNOS, IL-6, and TNF-α expression.	DSS-induced UC C57BL/6 mouse model.	(159)
	Anti-inflammatory effects.	↓NLRP3 expression *via* disruption of IL-17A signaling.	DSS-induced colitis C57BL/6 mice model.	(160)
	↓Lipid accumulation.	↓LXR-dependent SREBP-1c expression and intracellular lipid levels. ↓LXR-induced ABCA1 expression in Mø.	HepG2 cells and RAW264.7 Mø stimulated LXRα/β agonist (T0901317).	(161)
	Reparative effects of intestinal barrier injury.	↓MAPK/NF-κB/MLCK t activating indirectly Nrf2 signaling pathways.	Ethanol-induced intestinal barrier damage in a Caco-2 cell monolayer model.	(162)
Sesamin (*Sesamum indicum* seeds)	Anti-atherosclerotic effects: ↓oxLDL-elicited lipid accumulation and ↑ HDL-mediated cholesterol efflux.	↑ PPARγ-dependent ABCG1 mRNA levels.	RAW264.7 Mø stimulated with oxLDL and sesamin.	(163)
	Antioxidants and anti-inflammatory effects.	Cytoprotective effect *via* Glutathione-S-transferase (GSH)-mediated ROS scavenger.	Caco-2 cells stimulated by H_2_O_2_	(164)
	↓Cholesterol absorption by enterocytes. ↓ Hepatic lipogenic genes expression. Antagonist ligand of LXRα.	Nrf2/ARE signaling activation dependent on ERK and AKT activation. ↑LXR-induced ABCA1/G1 expression.	LS174T colonic epithelial cells with LXRα agonist (T090) treatment.	(165)
Curcumin (Turmeric)	Anti-inflammatory effects.	↓NLRP3 inflammasome activation.	DSS+LPS-stimulated peritoneal Mø and bone marrow-derived Mø (BMDMs).	(166)
	↑Cholesterol efflux.	miR-125a-5p/SIRT6 axis, ↑ ABCA1 expression.	THP-1 Mø treated with ox-LDL.	(167)
	↑Cholesterol efflux.	Dependent-Nrf2 activation HO-1 expression, regulating the expression of ABCA1 and SR-BI.	Raw264.7 and THP-1 Mø.	(168)
	↓Cholesterol uptake.	↓NPC1L1 expression by ↓SREBP2.	Cholesterol micelle-treated Caco-2 cells.	(169, 170)
	↓Severity of disease and ↓Colonic inflammation induced by TNBS.	↑PPAR-γ, ↓COX-2, and ↑ PGE2 and ↑15d-PGJ2 expression.	TNBS-induced colitis rats.	(171)
	Protective effects through preventing inhibition of mitochondrial respiration and restoring mitochondrial redox balance.	↑Mitochondrial respiration and complex I activity. Restores superoxide anion generation, mtGSH, mitochondrial nitrites, and aconitase activity.	TNBS-induced chronic colitis mice.	(172)
Polyphenolic maqui extract (Ach)	↓Damage signs, ↓Transmural inflammation, and ↑Mucosal architecture and its muco-secretory function.	Activation Nrf-2/HO-1 pathway. Regulation of COX-2 and iNOS.	TNBS-induced CD Balb/c mice.	(173)

↑ increase; ↓ decrease.

Nrf2 is a redox-sensitive transcription factor sequestered in the cytoplasm by Kelch-like ECH-associated protein1 (Keap1), remaining inactive under physiological conditions and being degraded by the proteasome (175). Upon oxidative stress, Nrf2 is released from Keap1, translocates to the nucleus, and forms a heterodimer with small Maf proteins (175). This heterodimer allows cellular protection and adaptive responses through the expression of phase II-detoxifying enzymes (glutathione S-transferase (GST), NAD(P)H: quinone oxidoreductase (NQO1)), stress-responsive proteins (heme oxygenase-1 (HO-1)), and ROS scavenging enzymes (glutathione peroxidase (GPx), superoxide dismutases (SOD) (175, 176) (**Figure 3**). Additionally, Nrf2 activation regulates mitochondrial biogenesis by activating nuclear respiratory factor-1 (NRF-1), transcribing mitochondrial transcription factor A (TFAM), and mitochondrial transcription factor B2 (TFBM2) and regulating PGC-1α expression (177). Interestingly, evidence suggests that down-regulation of Nrf2/PGC-1α axis contributes to susceptibility to inflammatory bowel disease (92, 178, 179), indicating that activation of Nrf2 signaling by dietary phytochemicals may alleviate oxidative stress and mitochondrial dysfunction in IBD.

In addition, phytochemicals, especially polyphenols, are players in the deacetylation process of PPAR-γ and PRDM16, causing the UCP1 overexpression and increase of PPAR-γ, PPAR-α, SIRT1, and PGC-1α expression (**Figure 4**). Moreover, *via* transient receptor potential vanilloid 1 (TRPV1) some phytochemicals can also increase the SIRT1 expression (180). In addition, other phytochemicals, such as quercetin, curcumin, and resveratrol can activate the β3-adrenergic pathway (β3-AR), increasing cAMP levels by activating the MAPKs pathway, resulting in TG hydrolysis, FA oxidation and increasing mitochondrial UCP1 transcription and activity (181). In DSS-treated mice, reduced *UCP1* expression in subcutaneous adipose tissues was found in conjunction with increased intestinal permeability (86). These findings could suggest a role in IBD pathogenesis or disease progression. However, no data about its expression in epithelial or lamina propria cells, nor its bioenergetics outcomes, has been addressed so far.

On the other hand, *LXR* expression is decreased in IBD (56, 57). LXR activation enhances the intestinal *ABCA1* and *ABCG1* expression while it reduces *NPC1L1* expression, resulting in increased intracellular cholesterol efflux and limiting *exogenous cholesterol* absorption by enterocytes (39, 182). Furthermore, plasma-derived cholesterol from the enterocyte basolateral membrane is excreted to the gut lumen (e.g., transintestinal cholesterol excretion) by ABCG5 and ABCG8, both of which are regulated by LXR (183, 184). Of note, in atherosclerosis, diabetes, and diabetic retinopathy using a SIRT1 agonist, LXR is deacetylated by SIRT1 at a specific conserved lysine (K432 in LXRα and K433 in LXRβ), thereby activating LXR (185–188) suggesting that SIRT1 may be a potential IBD target to promote LXR-mediated cholesterol efflux.

Hence, Nrf2-, PPAR-γ-, and LXR-mediated signaling pathway regulation by dietary phytochemicals enhance the antioxidant defense and reduce inflammation in both *in vitro* and *in vivo* IBD models (**Table 1**). Some phytochemicals such as luteolin, sesamin, and maqui extract have an unknown direct effect on mitochondrial dysfunction in IBD, becoming an interesting topic of study. Additionally, further studies of Nrf2, PPAR-γ, and LXR upstream pathways are required for a better understanding of the antioxidant, anti-arteriosclerotic, and anti-inflammatory effects of these dietary compounds.

## Conclusion and future perspectives

IBD is characterized by a loss of intestinal barrier function and inflammation. During IBD pathogenesis, trafficking of intracellular cholesterol is altered, with decreased cholesterol transporter expression, reducing cholesterol efflux and increasing influx. Since cholesterol is an important component of the plasma membranes and endomembranes (ER and mitochondria), abnormal cholesterol accumulation promotes ER stress and mitochondrial dysfunction during IBD. Furthermore, mitochondria in IBD are characterized by decreased ETC expression and activity, Δψm drop, mtROS production, and decreased TCA cycle intermediates, leading to a metabolic shift towards glycolysis as an adaptive response. Chronic inflammation during IBD may be mediated by NLRP3 inflammasome activation by DAMPs, such as cholesterol, mtROS, and mtDNA, generating an IL-1β-mediated pro-inflammatory response. Notably, the recovery of the mitochondrial function with correct cholesterol trafficking could be a potential therapeutic strategy for IBD. In addition, it has been discussed that the high prevalence and incidence in industrialized countries could be related to a Western diet, due to the high intake of saturated fatty acids, cholesterol, refined carbohydrates, and sugar may impact the inflammatory status becoming essential during disease pathogenesis (189). Therefore, changing towards an increase in the intake of whole grains, vegetables, fruit, seeds, and polyunsaturated fatty acids, with are part of a Mediterranean diet characterized by a higher content of phytochemicals and essential fatty acids could bring benefits to IBD patients. In this line, several dietary phytochemicals have been identified as efficient agents reducing inflammasome activation through the recovery of cholesterol efflux and mitochondrial function, which could reduce immunopathogenic effects in IBD patients.

The role of altered cholesterol trafficking and accumulation in IBD inflammation is an emerging area that offers new perspectives to understanding the pathogenic mechanisms of IBD. This may represent new opportunities for the discovery of drugs with mitochondrial functions that inhibit NLRP3 inflammasome activation.

## Author contributions

JA wrote most of the review. NG contributed to writing and made the figures. FU, KD, and MAH contributed to the writing and participated reviewing and critically correcting the manuscript, MAH corrected on manuscript structure and supervised the work. KN, MF, and GL participated reviewing and critically correcting the manuscript. All authors contributed to the article and approved the submitted version.

## Funding

This work was funded by the Agencia Nacional de Investigación y Desarrollo (ANID)/PhD fellowship #21220889 (JA), and #21200669 (NG), FONDECYT Grants #1220702 (MAH), #11201322 (FAU), MiBi: interdisciplinary group on mitochondrial targeting and bioenergetics ACT 210097 (FAU); FONDECYT postdoctoral grant #3210367 (KD-C).

## Acknowledgments

We wish to thank David Cox for his editing. Figures were created with BioRender.com.

## Conflict of interest

The authors declare that the research was conducted in the absence of any commercial or financial relationships that could be construed as a potential conflict of interest.

## Publisher’s note

All claims expressed in this article are solely those of the authors and do not necessarily represent those of their affiliated organizations, or those of the publisher, the editors and the reviewers. Any product that may be evaluated in this article, or claim that may be made by its manufacturer, is not guaranteed or endorsed by the publisher.
