# Corrigendum: The role of cholesterol and mitochondrial bioenergetics in activation of the inflammasome in IBD

**DOI:** 10.3389/fimmu.2024.1384162

**Published:** 2024-02-26

**Authors:** Jessica Astorga, Naschla Gasaly, Karen Dubois-Camacho, Marjorie De la Fuente, Glauben Landskron, Klaas Nico Faber, Félix A. Urra, Marcela A. Hermoso

**Affiliations:** ^1^ Laboratory of Innate Immunity, Program of Immunology, Institute of Biomedical Sciences, Faculty of Medicine, Universidad de Chile, Santiago, Chile; ^2^ Immunoendocrinology, Division of Medical Biology, Department of Pathology and Medical Biology, University Medical Center Groningen, Groningen, Netherlands; ^3^ Department of Gastroenterology and Hepatology, University Medical Center Groningen, Groningen, Netherlands; ^4^ Laboratory of Metabolic Plasticity and Bioenergetics, Program of Molecular and Clinical Pharmacology, Institute of Biomedical Sciences, Faculty of Medicine, Universidad de Chile, Santiago, Chile; ^5^ Laboratory of Biomedicine Research, School of Medicine, Universidad Finis Terrae, Santiago, Chile

**Keywords:** IBD - inflammatory bowel disease, intracellular cholesterol accumulation, mitochondrial dysfunction, inflammasome, NLRP3 inflammasome, diet phytochemicals

In the published article, there was an error in the legend for

**Table 1** Dietary phytochemicals and their restorative effects on mitochondrial function and lipid homeostasis as published. ↓ increase; ↑ decrease (on table footnotes). The corrected legend appears below.

**Table 1:** Dietary phytochemicals and their restorative effects on mitochondrial function and lipid homeostasis.

Phytochemical (Source)	Outcome	Proposed pathway	Model	Reference
Resveratrol (Grape, wine, peanut, and cranberry)	Anti-inflammatory protection and oxidative stress inhibition against intestinal inflammation. Anti-inflammatory effects. Protective effects against the alterations of mitochondrial function and oxidative stress. ↓Disease activity and ↑Quality of life in UC patients at least partially through the ↓Oxidative stress. Anti-atherosclerotic effects: ↓FA and MAG intestinal accumulation. Restoration of succinate and lactic acid levels.	Nrf2 activation: ↑Oxygenase-1 (HO-1) mRNA, ↓ROS production, and ↑PPAR-γ accumulation. ↑Nrf2, ↓IL-1β, and ↑IL-11. ↑Intracellular ATP, protective effects against ↓Δψm induced by INDO. - Abolishes oleate-triggered lipid, total cholesterol, and esterified cholesterol accumulation by activating PPAR-α and PPAR-γ signaling.	cytokine-stimulated (IL-1α, TNF-α, IFN-γ) HT-29 cells *In vitro* UC model in Caco-2 cells challenged with TNF-α. Intestinal epithelial Caco-2 cells induced by indomethacin (INDO). Prospective, randomized, double-blind, placebo-controlled study in UC patients. Supplements (containing 500 mg trans-resveratrol) or placebo capsules. *ApoE* null mice fed with a high-fat diet (HFD) (AS) and resveratrol intervention. RAW 264.7 mice Mø treated with oleate and resveratrol.	(147) (148) (149) (150) (151)
Quercetin (onion, apple grape, and citrus fruits)	Protective effects against the alterations of mitochondrial function and oxidative stress. Mitochondrial protective effects against and maintenance of gastrointestinal mucosal renewing regulating apoptosis. ↓NLRP3 inflammasome activation and ↓Mitochondrial damage. Intestinal anti-inflammatory effects via Nrf2/HO-1	↑Intracellular ATP, protective effects against ↓Δψm induced by INDO, and inhibition of the inhibitory effects of INDO and rotenone on complex I. Prevents Ca^2+^ mobilization induced by INDO and its consequences, including ↑Caspase-3 and caspase-9 activation and cytochrome C release. ↓Activity of caspase-1 and ↓Secretion of IL-1β and ↓IL-18 via NLRP3 inflammasome. Improvement in Δψm, blocking cytochrome C release, ↓O_2_ consumption, ↓ mtDNA cytosolic content, and ↓ ROS level. ↓TNF-α, IFN-γ, and IL-6. Nuclear Nrf2 accumulation ↑ HO-1 expression in colonic Mø.	Intestinal epithelial Caco-2 cells induced by INDO. Intestinal epithelial Caco-2 cells induced by INDO. Caco-2 cells infected by *Escherichia coli* O157:H7 T cell-dependent colitis model induced by the adoptive transfer of *naive* T cells into *Rag1* null mice and DSS-induced colitis mice model.	(149) (152) (153) (154)
Sulforaphane (cruciferous vegetables)	Antioxidant and anti-inflammatory effects, ↑ Mitochondrial bioenergetic function upon cholesterol-induced pancreatic β-cell dysfunction. ↓Intestinal permeability upon LPS, ↓Oxidative stress, ↓Inflammation, and ↓apoptosis.	Improving ATP turnover, spare capacity, and impairment of the electron flow at complexes I, II, and IV. ↓NFκB pathway. ↑SIRT1 and ↑PGC-1α expression. ↑ Antioxidant enzymes of the Nrf2 pathway and ↓Lipid peroxidation induced by cholesterol. Activating the AMPK/SIRT1/PGC-1 pathway.	Min6 cells, a β-cell line exposed to high concentration of cholesterol. LPS-induced Caco-2 *in vitro* model.	(155) (156)
Dried apple peel polyphenols (DAPP)	↓DSS-induced damage, ↓Pro-inflammatory factors, ↓Oxidative markers, and ↓ROS. ↓Mitochondria-dependent cell death, ↑β-oxidation, ↑Mitochondrial bioenergetics, and ↓Alteration in mitochondrial morphology.	↓TNF-α, COX-2, and iNOS.	*In vivo* model of DSS-induced colitis in male C57BL6 mice.	(157>)
Strawberry Ellagitannin-Rich Extract (S-ET)	↓ HFD effects in rats, ↓Body weight, ↓Relative mass of the epididymal pad, ↓Hepatic fat, ↓Oxidized glutathione, ↓TG, ↓Total cholesterol, and ↓Thiobarbituric acid-reactive substances concentrations and improve blood plasma parameters.	↓H_2_O_2_ and SOD2 protein expression and ↑8-oxoguanine DNA glycosylase 1 (OGG1) expression. ↑CPT-1 and ACADL, ATP production, and PGC-1α. ↓NF-kB and AP-1.	HFD supplemented with S-ET) in Male Wistar rats. *In vivo* model	(158)
Luteolin (carrot, pepper, celery, spinach, and parsley)	Antioxidants and anti-inflammatory effects. Anti-inflammatory effects. ↓Lipid accumulation. Reparative effects of intestinal barrier injury.	↑ Nrf2 and ↓iNOS, IL-6, and TNF-α expression. ↓NLRP3 expression via disruption of IL-17A signaling. ↓LXR-dependent SREBP-1c expression and intracellular lipid levels. ↓LXR-induced ABCA1 expression in Mø. ↓MAPK/NF-κB/MLCK t activating indirectly Nrf2 signaling pathways.	DSS-induced UC C57BL/6 mouse model. DSS-induced colitis C57BL/6 mice model. HepG2 cells and RAW264.7 Mø stimulated LXRα/β agonist (T0901317). Ethanol-induced intestinal barrier damage in a Caco-2 cell monolayer model.	(159) (160) (161) (162)
Sesamin (*Sesamum indicum* seeds)	Anti-atherosclerotic effects: ↓oxLDL-elicited lipid accumulation and ↑ HDL-mediated cholesterol efflux. Antioxidants and anti-inflammatory effects. ↓Cholesterol absorption by enterocytes. ↓ Hepatic lipogenic genes expression. Antagonist ligand of LXRα.	↑ PPARγ-dependent ABCG1 mRNA levels. Cytoprotective effect via Glutathione-S-transferase (GSH)-mediated ROS scavenger. Nrf2/ARE signaling activation dependent on ERK and AKT activation. ↑LXR-induced ABCA1/G1 expression.	RAW264.7 Mø stimulated with oxLDL and sesamin. Caco-2 cells stimulated by H_2_O_2_ LS174T colonic epithelial cells with LXRα agonist (T090) treatment.	(163) (164) (165)

[↑ increase; ↓ decrease]

[↑ increase; ↓ decrease]

In the published article, there was an error in the Funding statement. This work was funded by the Agencia Nacional de Investigación y Desarrollo (ANID)/PhD fellowship #21220889 (JA), and #21200669 (NG), FONDECYT Grants #1120702 (MAH), #11201322 (FAU), MiBi: interdisciplinary group on mitochondrial targeting and bioenergetics ACT 210097 (FAU); FONDECYT postdoctoral grant #3210367 (KD-C). The correct Funding statement appears below.

**FUNDING**

This work was funded by the Agencia Nacional de Investigación y Desarrollo (ANID)/PhD fellowship #21220889 (JA), and #21200669 (NG), FONDECYT Grants #1220702 (MAH), #11201322 (FAU), MiBi: interdisciplinary group on mitochondrial targeting and bioenergetics ACT 210097 (FAU); FONDECYT postdoctoral grant #3210367 (KD-C).

The authors apologize for these errors and state that this does not change the scientific conclusions of the article in any way. The original article has been updated.

